# Paraoxonase 1 Gene Polymorphism Does Not Affect Clopidogrel Response Variability but Is Associated with Clinical Outcome after PCI

**DOI:** 10.1371/journal.pone.0052779

**Published:** 2013-02-13

**Authors:** Kyung Woo Park, Jin Joo Park, Jeehoon Kang, Ki-Hyun Jeon, Si-Hyuck Kang, Jung-Kyu Han, Sang Eun Lee, Han-Mo Yang, Hae-Young Lee, Hyun-Jae Kang, Bon-Kwon Koo, Byung-Hee Oh, Young-Bae Park, Hyo-Soo Kim

**Affiliations:** Department of Internal Medicine and Cardiovascular Center, Seoul National University Hospital, Seoul, Korea; Vanderbilt University, United States of America

## Abstract

**Background:**

Paraoxonase (PON) is a high-density-lipoprotein (HDL) associated enzyme with antioxidative and anti-atherogenic property. Its function is associated with coronary artery disease and its activity genetically controlled. We evaluated whether genetic variation of PON-1 is associated with clinical outcome in a large cohort of Korean patients with drug-eluting stents implantation.

**Methods:**

A total of 1676 patients with drug-eluting stent implantation were enrolled in the prospective CROSS-VERIFY cohort from June 2006 to June 2010. We genotyped the PON1-Q192R gene, measured clopidogrel on-treatment platelet reactivity (OPR), and analyzed lipid profiles. The primary endpoint was the composite of cardiac death, myocardial infarction, and stent thrombosis at 12 months.

**Results:**

PON-1 genotyping data were available in 1336 patients. Since the Q-allele is associated with decreased PON-activity, we analyzed the outcome between patients with QQ/QR (815 patients, 61%) and those with RR-genotype (521 patients, 39%). After adjustment for common cardiac risk factors, the QQ/QR-genotype was an independent predictor of the primary thrombotic endpoint with an 11-fold increased risk (HR 11.6, 95% CI: 1.55–87.0), but not repeat revascularization (HR 1.12, 95% CI: 0.78–1.61). The QQ/QR-genotype was not associated with OPR (QQ/QR: 231±86 PRU vs. RR 236±82 PRU, p = 0.342) but higher small-dense LDL levels (1.20±0.12 mg/dL vs. 0.76±0.15 mg/dL, p = 0.027). The increased risk of thrombotic outcomes was more profound in acute coronary syndrome (ACS) patients compared with non-ACS patients.

**Conclusion:**

PON1 Q-allele is an independent predictor of worse cardiovascular outcome independent of platelet function and is associated with significantly higher levels of small dense LDL-C.

## Introduction

Paraoxonase (PON) is a calcium-dependent, multifunctional antioxidant enzyme that is widely distributed [1]. The activity of PON is genetically controlled, and the Q192R single-nucleotide polymorphism (SNP) in the paraoxonase PON-1 gene is the best known genetic determinant of PON-activity [2]. Recently, this genetic variation was reported to be strongly linked to clopidogrel bioactivation, clopidogrel on-treatment platelet reactivity (OPR), and thrombotic clinical outcome [3]. However, subsequent studies failed to reproduce the association between PON-1 polymorphism and clopidogrel response variability [4]–[7]. Others have suggested that PON-1 genotype may be associated with long term clinical outcome in patients with known CAD possibly due to antioxidant effects on low density lipoprotein-cholesterol (LDL-C) [8].

This study was undertaken to elucidate whether PON-1 genetic variation is associated with clinical outcome after percutaneous coronary intervention (PCI) with drug-eluting stents (DES). We further tried to dissect the mechanism by which PON-1 genetic variation could possibly affect clinical outcome by evaluating the relationship between PON-1 SNP and clopidogrel response variability and lipid parameters including LDL-C particle size.

## Methods

### Study Population

The CROSS-VERIFY cohort (measuring clopidogrel resistance to assure safety after percutaneous coronary intervention using VerifyNow) is a dynamic cohort with on-going patient recruitment including consecutive patients receiving PCI who agreed to measurement of clopidogrel OPR with the VerifyNow P2Y12 assay after clopidogrel therapy at Seoul National University Hospital since June 1^st^ 2006. The enrollment period for the current study was from June 2006 through June 2010, and only patients with implantation of drug eluting stent (DES) were enrolled. Exclusion criteria were contraindication to aspirin, clopidogrel, or heparin; the use of intravenous glycoprotein IIb/IIIa inhibitor within 5 days before the platelet reactivity test; the concomitant use of cilostazol; uncontrolled malignancy; bleeding tendency; and ethnicity other than Korean heritage. The study complied with Declaration of Helsinki and was approved by the Institutional Review Board (IRB) of Seoul National University Hospital. The participants provided their written informed consent to participate in this study. The ethics committee approved the consent procedure.

### Platelet Function Test

The inhibitory effect of clopidogrel on platelet reactivity was measured using the VerifyNow P2Y12 assay (Accumetrics Inc., San Diego, CA). Blood sample was obtained 12 to 24 hours after final dose of clopidogrel in patients who had been on 75 mg of clopidogrel for more than 7 days, and 12 to 24 hours after PCI in patients who were loaded with clopidogrel before catheterization. A loading dose of 300 mg clopidogrel was administered in patients who had been taking clopidogrel for less than 7 days; 600 mg was given to clopidogrel-naïve patients. All patients took aspirin at 100 mg per day or 300 mg loading, if not taken previously. The VerifyNow P2Y12 assay reports the results as P2Y12 reaction units (PRU).

### Lipid Profile and LDL-C Particle Size Measurement

Fasting blood samples were obtained by venipuncture on the day of the PCI. Serum was separated by centrifugation and biochemical measurements were conducted immediately. Serum total cholesterol (TC), LDL-C, high-density lipoprotein cholesterol (HDL-C), and triglyceride (TG) were measured enzymatically using the Hitachi 747 chemical analyzer (Hitachi, Tokyo, Japan). LDL particle size was determined using gel electrophoresis (LipoprintTM System; Quantimetrix Corp., Redondo Beach, CA, USA) according to the manufacturer’s instructions [9].

### Genetic Analysis

PON-1 (Q192R, rs662) was screened using the TaqMan fluorogenic 5′ nuclease assay (ABI, Foster City, CA). The PCR was performed using 384-well plates by a Dual 384-Well GeneAmp PCR System 9700 (ABI, Foster City, CA) and the endpoint fluorescent readings were performed on an ABI PRISM 7900 HT Sequence Detection System (ABI, Foster City, CA). Duplicate samples and negative controls were included to ensure accuracy of genotyping.

### Endpoints

The primary endpoint was the composite of cardiac death, non-fatal myocardial infarction (MI), and stent thrombosis at 12 months. Secondary endpoints included each individual components of the primary end point, target lesion revascularization (TLR) at 12 months, and clopidogrel OPR. All deaths were considered to be cardiac deaths unless an unequivocal non-cardiac cause could be documented. Myocardial infarction was defined according to the universal definition of myocardial infarction [10]. Stent thrombosis was defined according to the Academic Research Consortium definition, including definite and probable stent thrombosis [11].

### Follow-up

All patients had regular out-patient visits with a cardiology specialist. Data were prospectively gathered by a dedicated coordinator. When follow-ups were missed or key data were missing, telephone contacts were made by data coordinators. To confirm the completeness of follow-up and minimize underreporting of events, the vital status of all patients were checked with the National Statistical Agency and events of patients with possible follow-up loss with the National Health Insurance Corporation.

### Statistical Analysis

The data were presented as numbers and frequencies for categorical variables, and as the mean ± the standard deviation for continuous variables. For comparison among groups, the Chi-square test (or Fisher’s exact test when any expected cell count was <5 for a 2×2 table) was used for categorical variables and the unpaired Student’s t-test or the 1-way analysis of variance were applied for continuous variables. The Chi-square test for goodness of fit was used to verify agreement with Hardy-Weinberg equilibrium using the Fisher’s exact test. The chronological trend of outcomes was expressed as Kaplan–Meier estimates at 12 months and was compared between PON-1 QQ/QR- and RR-genotype carriers. The log-rank test was used to analyze the significant indifferences in clinical outcomes. A multivariable Cox proportional hazards regression model was used to find the independent predictors of thrombotic events after PCI. The analysis of covariance (ANCOVA) was applied to quantify the effect of PON-1 Q192R genotypes on LDL-particle size by entering patients’ genotype status as factor and clinical factors as covariates. In this case, the data were presented as the mean ± the standard error. Two sided p values less than 0.05 were considered statistically significant. Statistical tests were performed using SPSS version 17 (SPSS Inc., Chicago, Illinois, USA).

## Results

### Study Population

A total of 1676 patients were enrolled in the CROSS-VERIFY Cohort for the current study. We excluded 38 patients without drug eluting stent implantation, 143 patients with concomitant use of cilostazol, 2 Caucasians, and 157 patients who did not agree with genetic analysis or failed PON-1 Q192R genotyping, leaving 1336 patients available for the current analysis.

PON1-genotyping was successful in 98%. The observed genotype distribution was 13.4%, 47.6% and 39% for QQ, QR and RR-genotype, respectively. The genotyping replication rate for PON Q192R was 99%. The minor allele frequency was 0.37 and the genotype distribution was within Hardy-Weinberg-Equilibrium (*X* ^2^, P* = *0.490). The baseline characteristics of the study population are summarized in **Table S1**. The mean age was 64 years old, 68% were male, 68% had hypertension, and 32% had diabetes mellitus. Seven hundred and seventy-three patients (57.9%) presented with stable angina, while 563 patients (42.1%) with acute coronary syndrome (ACS). When grouped according to genotype, there was no difference in baseline characteristics among the three groups. There was no relationship between the distribution of the paraoxonase 1 gene polymorphism and clinical presentation (P = 0.418).

### Clinical Outcomes

**Figure 1:**
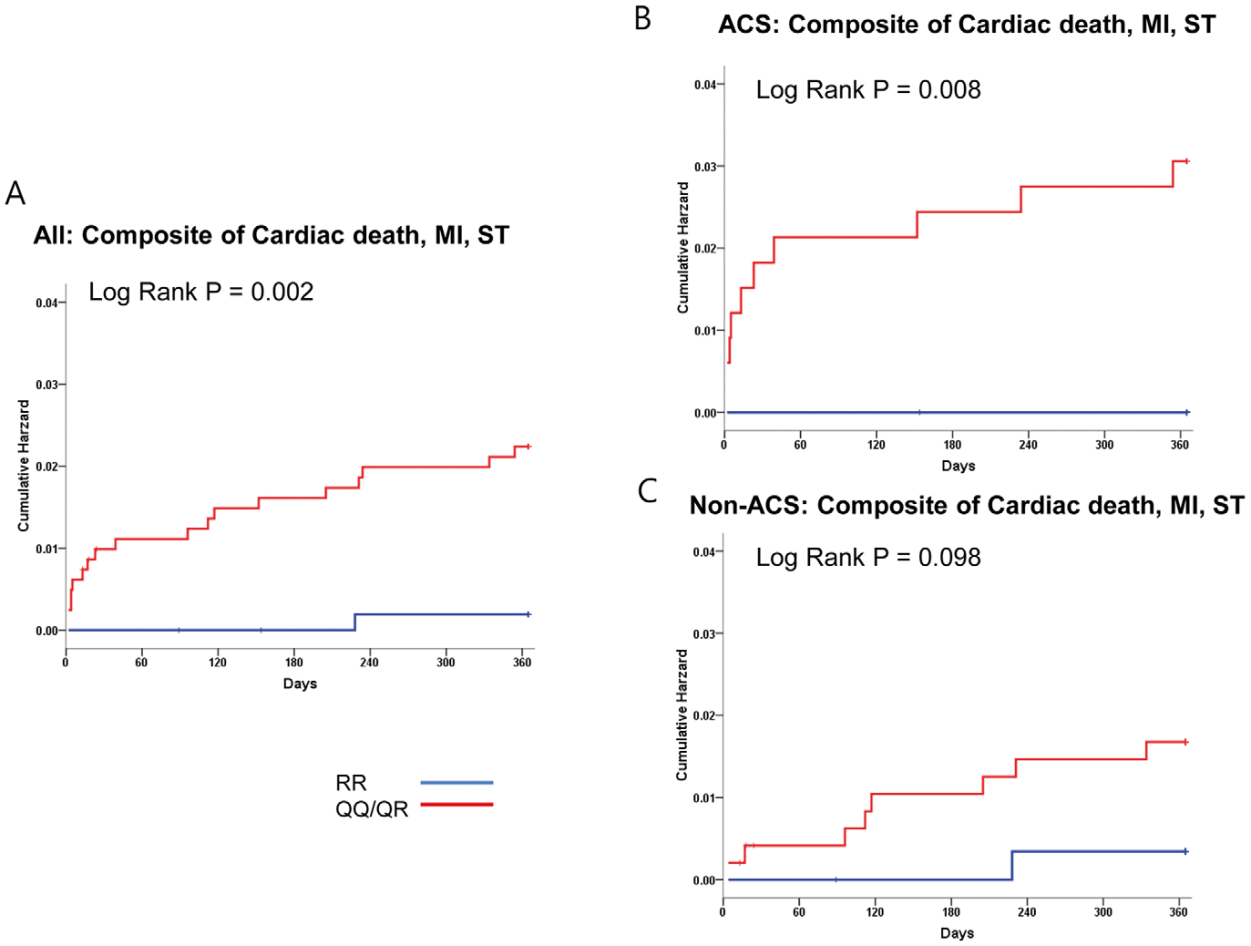
Kaplan-Meier curves for primary composite end point of cardiac death, myocardial infarction, and stent thrombosis. Patients with PON-1 QQ/QR genotypes had higher rates for primary endpoint than those with RR-genotype (2.2% vs. 0.2%, Log-rank p = 0.002) (**Fig. 1A**). This difference in thrombotic outcomes was more profound in the higher risk ACS patients compared with the lower risk non-ACS patients (3% vs. 0%, Log-rank p = 0.008) (**Fig. 1B**), although the trend toward higher risk of thrombotic outcome in the QQ/QR group was maintained even in the non-ACS group (1.7% vs. 0.3%, Log-rank p = 0.098) (**Fig. 1C**).

**Figure 2:**
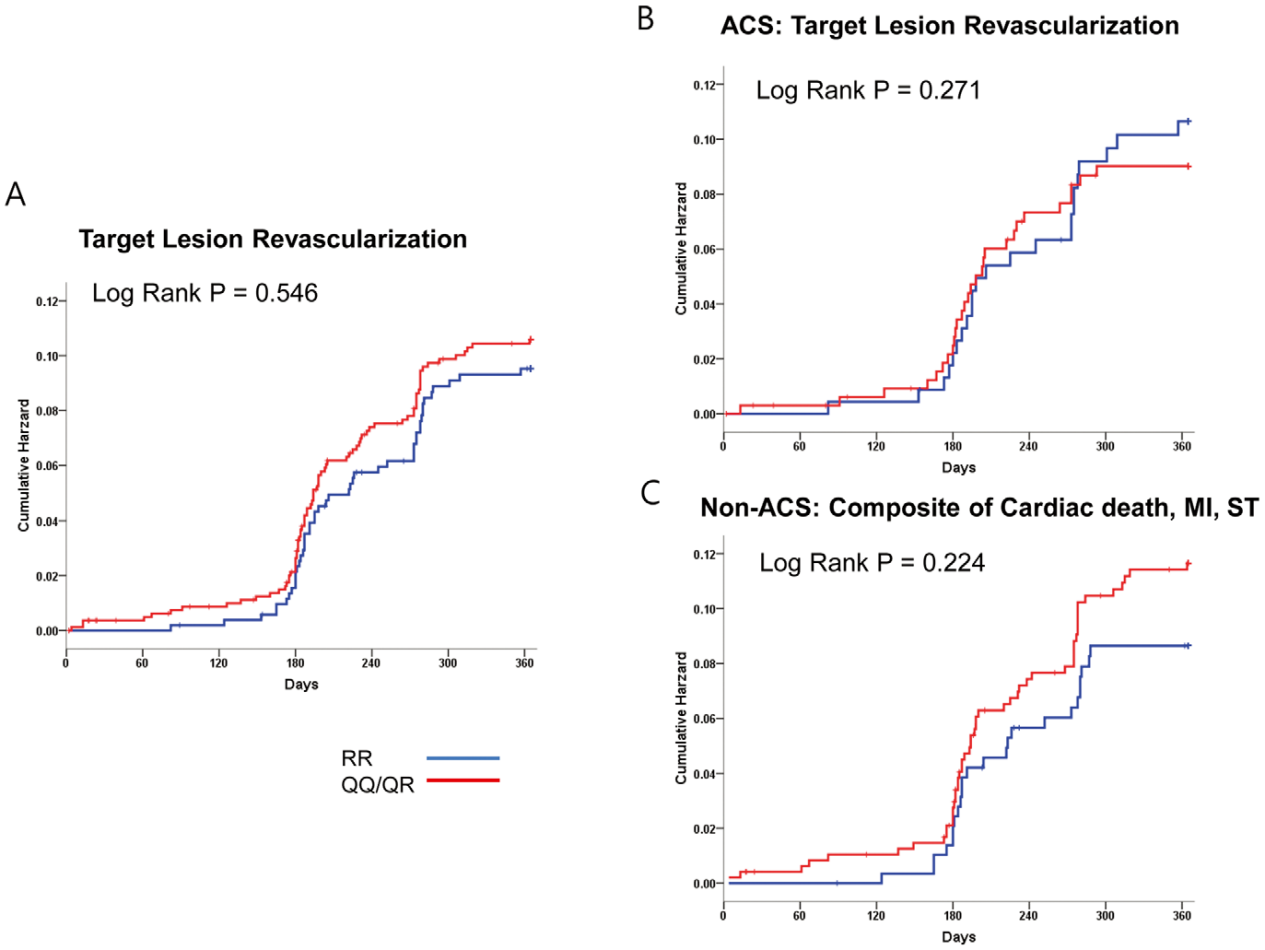
Kaplan-Meier curves for target lesion revascularization. There was no difference in target lesion revascularization rates between PON-1 QQ/QR and RR genotypes either in all patients (9.8% vs. 9.0%, Log-rank P = 0.546) (**Fig. 2A**), in those with ACS presentation (8.4% vs. 10%, Log rank P = 0.271) (**Fig. 2B**), or those with non-ACS-presentation (10.8% vs. 8.2%, Log-rank P = 0.224) (**Fig. 2C**).

The primary composite endpoint occurred in 1.4% (19 events during the one year follow-up). The baseline characteristics between the both groups with and without clinical events were mostly similar, except that the frequency of QQ/QR genotype was higher in the event group (60.5% vs. 94.7%, P = 0.002) and that of beta-blocking agents higher in the event-free group (51.5% vs. 26.3%, P = 0.029) (**Table 1**). Cardiac death occurred in 9 patients (0.7%), MI in 11 patients (0.8%), and stent thrombosis in 8 patients (0.6%). Since PON-Q allele is associated with decreased PON activity, and with similar clinical outcome (**Figure S1, Table S3**), we divided the patients into those with and without the Q-allele. Eight hundred and fifteen patients had QQ or QR genotypes (61%), while 521 patients had RR genotype (39%). The primary composite endpoint (2.2% vs. 0.2%, P = 0.002) as well as the individual endpoints of MI (1.3% vs. 0.3, P = 0.008) and stent thrombosis (1.0% vs. 0%, P = 0.023) occurred more frequently in patients with the QQ/QR genotype compared with those with the RR genotype (**Figure 1A**) (**Table 2**). This difference in thrombotic outcomes was more profound in the ACS patients compared with the non-ACS patients, although the trend toward higher risk of thrombotic outcome in the QQ/QR group was maintained even in the non-ACS group (**Figure 1B, 1C**). As for repeat revascularization, there was no difference in the risk of TLR between QQ/QR versus the RR genotypes (9.8% vs. 9.0%, P = 0.590) (**Figure 2A**), which was similar in both ACS and non-ACS subgroups (**Figure 2B, 2C**).

**Table 1:** Baseline characteristics according to primary endpoint.

	Event (−) N = 1317	Event (+) N = 19	p-value
**Demographic characteristics**			
Mean OPR (PRU)	233±83	245±89	0.535
PON-1 QQ/QR-genotype (%)	60.5	94.7	0.002
Age (years)	64±9	67±10	0.111
Men (%)	67.4	78.9	0.284
Body mass index (kg/m^2^)	25.2±4.9	24.1±3.9	0.355
Current smoker (%)	17.7	10.5	0.555
Hypertension (%)	67.7	63.2	0.678
Diabetes mellitus (%)	32.4	15.8	0.123
Dyslipidemia (%)	45.4	47.4	0.865
Chronic kidney disease (%)	2.4	5.3	0.380
- Renal replacement tx.	1.0	0	>0.999
Presenting symptoms (%)			0.503
- Stable angina	58	47.4	0.351
- Unstable angina	35.2	42.1	0.529
- NSTEMI	4.7	10.5	0.238
- STEMI	2.1	0	>0.999
Previous PCI (%)	32.3	36.8	0.672
Previous CABG (%)	3.0	5.3	0.449
Previous MI (%)	4.6	10.5	0.219
Congestive heart failure (%)	0.8	0	>0.999
Cerebrovascular accident (%)	5.8	5.3	>0.999
Peripheral artery disease (%)	1.2	0	>0.999
**Laboratory finding**			
GFR (ml/min/1.73 m^2^)	69.3±17.3	66.2±20.2	0.442
Cholesterol (mg/dL)	156±40	155±35	0.902
- Triglyceride (mg/dL)	143±94	128±69	0.512
- HDL-C (mg/dL)	42±12	42±13	0.765
- LDL-C (mg/dL)	88	87	0.910
**Concomitant Medication**			
ACEi/ARB (%)	41.9	52.6	0.345
Beta-blocker (%)	51.5	26.3	0.029
Calcium channel blocker (%)	26.9	26.3	0.956
-Dihydropyridine CCB (%)	16.9	15.8	0.895
-Non-DHP CCB (%)	10.2	15.8	0.434
Statin (%)	62.6	52.6	0.371
- Lipophilic statin (%)	41.4	42.1	0.949
Proton pump inhibitor (%)	2.2	10.5	0.070
- Omeprazol (%)	0.8	0	>0.999

ACEi, angiotensin converting enzyme inhibitor; ARB, angiotensin receptor blocker; CABG, coronary artery bypass graft; CCB, calcium channel blocker; HDL, high density lipoprotein; LDL, low density lipoprotein; OPR, on treatment platelet reactivity; PCI, percutaneous coronary intervention; PRU, P2Y12 reaction unit.

**Table 2:** Clinical outcome according to PON1-Q192R genotype.

	QQ/QR, n = 815	RR, n = 521	p-value
**Primary composite end point of cardiac death, MI, and ST**	18 (2.2%)	1 (0.2%)	0.002
**Secondary end points**			
Cardiac Death	8 (1.0%)	1 (0.2%)	0.085
Myocardial infarction	11 (1.3%)	0 (0.0%)	0.008
CVA	1 (0.1%)	0 (0%)	0.422
Stent thrombosis	8 (1.0%)	0 (0.0%)	0.023
Target lesion revascularization	80 (9.8%)	47 (9.0%)	0.590
Composite of Death, MI, CVA	16 (2.3%)	4 (0.6%)	0.002
MACE	113 (13.9%)	54 (10.4%)	0.052

MI, myocardial infarction; ST, stent thrombosis; CVA; cerebrovascular accident; MACCE, major adverse cardiovascular event.

P-value by Log-rank test.

To find the independent predictors of clinical outcome, a multivariate Cox-proportional hazard analysis was performed by entering common cardiac risk factors such as age (in decade), diabetes mellitus, dyslipidemia, and chronic kidney disease, along with PON-1 genotype. The QQ/QR genotype was an independent predictor of the primary endpoint with an 11-fold increased risk (HR 11.6, 95% CI: 1.55–87.0) along with age (age in decade HR 1.82, 95% CI: 1.08–3.07) (**Table 3**). As for repeat revascularization, diabetes mellitus (HR 1.51, 95% CI: 1.06–2.16), and dyslipidemia (HR 1.49, 95% CI: 1.04–2.12) were independent predictors, but not PON-QQ/QR genotype (HR 1.12, 95% CI: 0.78–1.61).

**Table 3:** Independent predictors of clinical outcomes.

	HR	CI	p-value
**Primary end point (composite of cardiac death, MI, Stent thrombosis)**			
- PON-QQ/QR0	11.6	1.55–87.0	0.017
- Age (in decade)	1.82	1.08–3.07	0.025
- Diabetes mellitus	0.40	0.12–1.34	0.146
- Dyslipidemia	1.23	0.50–3.04	0.655
- Chronic kidney disease	0.82	0.28–2.39	0.720
**Target lesion revascularization**			
- Diabetes mellitus	1.51	1.06–2.16	0.023
- Dyslipidemia	1.49	1.04–2.12	0.028
- Chronic kidney disease	1.37	0.92–2.04	0.117
- PON-QQ/QR	1.12	0.78–1.61	0.542
- Age (in decade)	0.97	0.78–1.18	0.772

Cox-proportional hazard regression analysis adjusting for PON-192 QQ/QR, age, diabetes mellitus, dyslipidemia, chronic kidney disease as covariates.

### PON-1 Genotype, Platelet Function and Lipid-profiles

**Figure 3:**
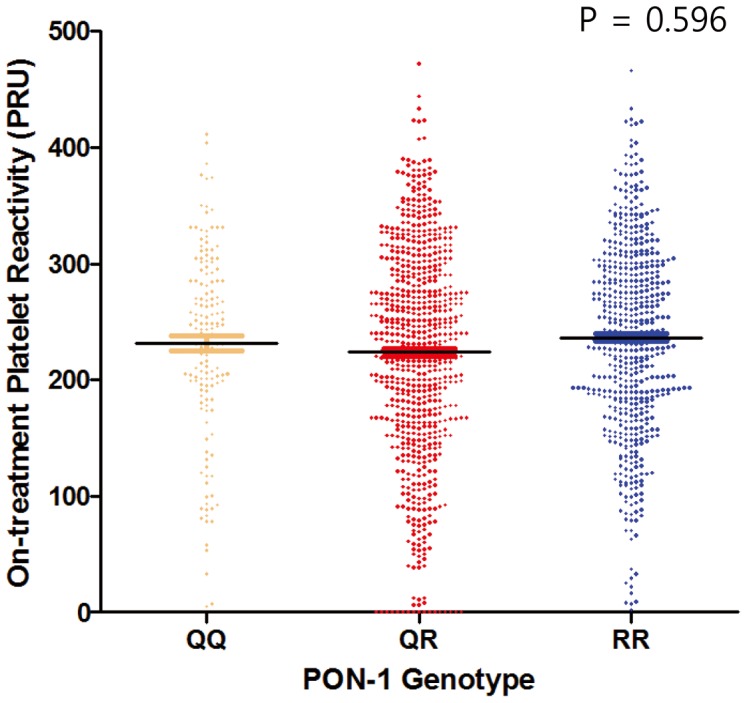
On-treatment platelet reactivity (OPR) according to PON1-Q192R genotypes. There was no difference in OPR between QQ, QR, and RR genotypes (mean OPR: 233±82 PRU, 231±86 PRU, and 236±81 PRU, for QQ, QR, and RR genotypes respectively, ANOVA P = 0.596).

There was no difference in both the distribution of platelet reactivity and mean OPR among the three genotypes (unadjusted mean OPR: 233±82 PRU, 231±86 PRU, and 236±81 PRU, for GG, GA, and AA genotypes respectively, ANOVA P = 0.596) (**Figure 3**). In addition, there was no difference in platelet reactivity according to the clopidogrel dosing regimen and the three genotypes (**Table S5**).

After adjustment for age (in decade), gender, smoking status, diabetes mellitus, chronic kidney disease, and dyslipidemia, there were no significant differences in OPR within the PON1-genotypes (adjusted mean OPR: 233±6, 231±3, and 234±4 PRU, for QQ, QR, and RR genotypes respectively).

**Figure 4:**
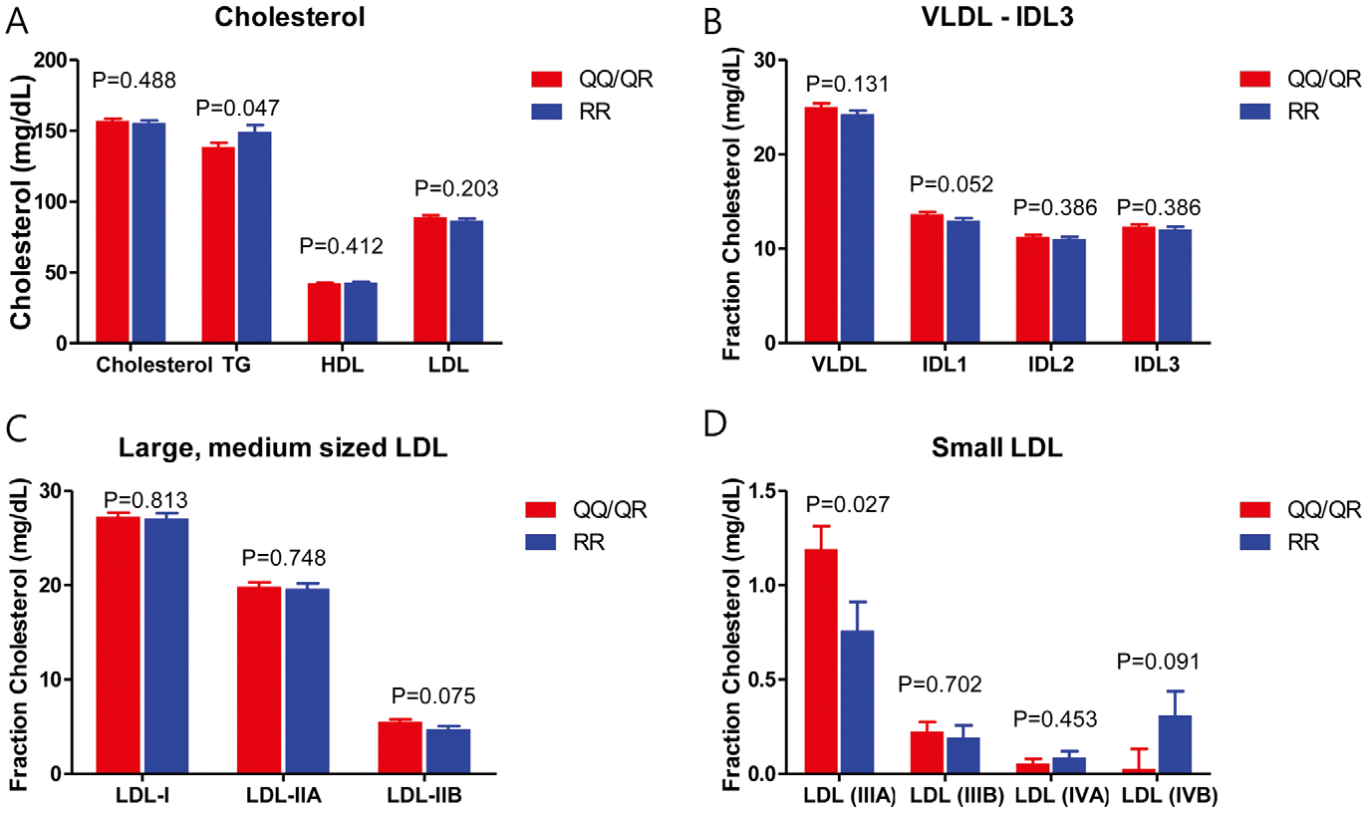
Lipid profiles according to genotypes. Patients with QQ/QR genotypes had lower triglyceride levels (**Fig. 4A**). As for LDL particle size, there was no difference either in VLDL, IDL between both groups (**Fig. 4B**), or large and medium sized LDL (**Fig. 4C**). As for small LDL, patients with QQ/QR genotype had significant higher LDL-IIIA than those RR-genotype (1.20±0.12 mg/dL vs. 0.76±0.15 mg/dL, P = 0.027) (**Fig. 4D**). Cholesterol level was compared by using one-way ANOVA test. As for LDL-particle size, general linear model analysis was applied to quantify the effect of PON-1 QQ/QR genotype on LDL-particle size after adjustment for age, sex, triglyceride level, HDL-C level, and statin use. Data were presented as the mean ± the SEM.

As for the relationship between PON-1 genotype and lipid profile, 63% of patients were on statin therapy, and the serum levels of total cholesterol, TG, HDL-C, and LDL-C in the entire patient cohort were 156±40 mg/dL, 143±94 mg/dL, 43±12 mg/dL, and 88±36 mg/dL, respectively. The baseline lipid parameters were mostly similar between the QQ/QR versus RR genotypes except for TG which was marginally higher in the RR genotype group (139±82 mg/dL vs. 149±110 mg/dL, P = 0.047). A total of 768 patients (57%) underwent LDL-particle size analysis. After adjustment for age, sex, TG, HDL-C, and statin use, patients with QQ/QR genotype had significantly higher small-dense LDL levels than those with RR-genotype (1.20±0.12 mg/dL vs. 0.76±0.15 mg/dL, P = 0.027) (**Figure 4**) (**Table S4**). As for the larger particle size LDL-C, i.e. LDL I-IIB, there was no significant difference between the two genotype groups.

## Discussion

To the best of our knowledge, this is the first study, which investigated the relationship between PON-1 genetic variants and clinical outcome in an exclusive cohort of patients who received DES. We report that PON-1 Q-allele is associated with increased risk for composite thrombotic outcome of cardiac death, MI and stent thrombosis, but not with increased risk for TLR. Patients with QQ/QR genotype have 11-fold increased risk for thrombotic complications than those with RR-genotype. However, QQ/QR genotype was not associated with clopidogrel response variability but with higher small dense (sd)-LDL level.

### PON-1 Q192R Genotype and Cardiovascular Outcome

The association between PON-1 Q192R genotypes and clinical outcome in coronary artery disease (CAD)-patients has been investigated in a couple of aspects. A cross-sectional genetic-association meta-analysis by Wheeler et al [12] involving 23,998 cases revealed no or minimal association between Q192R polymorphism and CAD. Reny et al also reported in a meta-analysis with 5,302 patients that he PON1-Q192R polymorphism has no major impact on the risk of MACE in clopidogrel-treated patients. [13].

In contrast, Mackness et al [14] showed that lower PON activity was associated with an increased risk for cardiovascular events in males, which was more pronounced in those with pre-existing CAD. The positive association between PON-1 genetic variation and clinical outcome in CAD-patients has been reported for long-term cardiovascular mortality and morbidity in 793 statin treated CAD patients in the REGRESS study [8]. In addition, Bhattacharyya et al [15] showed that those with the QQ-genotype were at increased risk for major adverse cardiovascular events. In line with these previous studies, our study comprised of patients receiving DES showed that the Q-allele carriers (PON-1 192QQ/QR genotypes) had an 11-fold risk increase for composite endpoint of cardiac death, MI and stent thrombosis. The association between PON-1 genotype and cardiovascular outcome was more profound in patients with ACS.

In contrast, we could not find any association between TLR and PON-1 genotypes. Chen et al [16] and Turban et al [17] also reported that Q192R genotype were not associated with severity, progression or regression of coronary atherosclerosis.

### Potential Mechanisms

Previously, Bouman et al reported that PON-1 Q192R was strongly linked to clopidogrel metabolism, and with clinical outcome including stent thrombosis, identifying the PON enzyme activity as a key step for clopidogrel bioactivation. This could be an important mechanism by which Q allele carriers are at increased risk for cardiovascular events. However, this cannot explain the association between PON-1 genotype and clinical outcome in many patients who were not taking clopidogrel in previous studies. Furthermore, subsequent studies by Sibbing et al, Fontana et al, Trenk et al, and Lewis et al could not reproduce the association between PON-1 polymorphism and clopidogrel response variability [4]–[6], [18]. Likewise, our results did not show any association between PON-1 Q192R polymorphism and mean OPR, either. Even the distribution of platelet function was very similar for the three genotype groups, suggesting mechanisms other than the effect on clopidogrel metabolism may be responsible for the different clinical outcome.

The first report on the genetic regulation of PON activity suggested that PON activity was controlled by two alleles at a single autosomal locus [19]. Later, Adkins et al identified that the serum paraoxonase-phenotype is associated with the ARG/Gln structural polymorphism at amino acid position 192 [2]. The 192R-alloenzyme was shown to bind to the HDL particle with 3-fold higher affinity than 192Q-alloenzyme, consequently exhibiting increased stability [20], and individuals with PON-1 192R-allele have increased PON activity. [3], [21] HDL-associated PON-1 (i) increases the reverse cholesterol transport, (ii) endothelial nitric oxide (NO) production, and (iii) promotes pronounced systemic antioxidant effects.

In this study, we found that patients with PON-1 QQ/QR genotypes had significantly higher sd-LDL-C level compared to those with RR-genotype, in particular LDL-IIIA. There are seven major LDL-subfractions (I (large), IIA and IIB (medium), IIIA and IIIB (small), and IVA and IVB (very small)). Predominance of LDL with small particle size has been associated with increased cardiovascular risk [22], and has been accepted as an emerging cardiovascular risk factor by the National Cholesterol Education Program Adult Treatment Panel III [23]. Small dense-LDL resides longer in plasma, has enhanced oxidizability, arterial proteoglycan binding and permeability through the endothelial barrier [24]. We hypothesize that impaired PON activity may lead to increased level of more atherogenic and *rupture-prone* sd-LDL-C resulting in thromboembolic events.

Another possible mechanism may be the endothelial dysfunction caused by decreased PON-1 activity. HDL-associated PON-1 activity plays a crucial role in maintaining the endothelial-atheroprotective effects of HDL. It stimulates the endothelial NO-production. NO has important antithrombotic properties, i.e. inhibiting leukocyte adhesion, limiting platelet adhesion and aggravation, and the expression of plasminogen activator inhibitor-1 [25]. Impaired HDL-associated PON-1 activity leads to decreased endothelial nitric oxide synthase (eNOS)-activity. Reduced endothelial NO bioavailability contributes to impaired endothelial repair, to the development and progression of atherosclerosis. Besler et al demonstrated that HDL from PON-1-deficient mice failed to stimulate NO production [26]. Patients with decreased paraoxonase activity, i.e. those with 192Q-allele, may have decreased HLD-associated anti-inflammatory and anti-thrombotic effect. Consequently, they would be at increased risk for thromboembolic complication. Besler et al also demonstrated that HDL from healthy subjects stimulated endothelial NO production, whereas that from stable CAD patients did not. Interestingly, HDL from ACS patients even inhibited the endothelial NO production. We showed that (i) 192Q-allele carriers had higher risk for thrombotic complication, and that (ii) this risk was more pronounced in ACS patients. Taken together, ACS patients are more susceptible to thrombotic complication due to HDL-PON mediated inhibition of endothelial NO synthesis, and this effect is exaggerated by the low PON activity due to PON-1 192Q-alleles.

With regard to HDL-C level, there are conflicting results on the association between PON Q192R genotype and serum HDL-C levels. In our study we could not demonstrate any association. In a study by Srinivasan et al [27] with 1,786 black and white young adults, the frequency of the PON-1 R-allele was higher in blacks than in whites (0.668 versus 0.297, P<0.001), and the R-allele was associated with increased HDL-C in whites (P = 0.041), whereas the opposite was true in blacks (P = 0.008). Ethnicity might play a role in differences in phenotypic expression of PON1 genotype on lipid levels.

### The Frequency of PON-1 Q192R Genotypes in Koreans

It is essential to determine first whether the differences in genotype frequencies observed in our study are attributed to the differences due to ethnicity or due to selection of patients with PCI-phenotype. Two studies reported the frequencies of PON Q192R-genotypes in healthy Koreans. In a study by Eom et al [28] the frequency of QQ, QR and RR genotypes was 13%, 42% and 45%, respectively, and in another study by Lee et al [29], it was 6.2%, 45.2%, and 41.2%, respectively. These study results suggest that the distribution of Q and R alleles is inverted in Koreans compared with Caucasians, and the low Q-allele frequency in our study population is not due to the selection of a cohort with PCI-phenotypes.

If the PON 192Q-allele is associated with increased risk for atherosclerotic burden, it is likely that the frequency of the 192 Q–allele is higher in the PCI cohort than the general Korean population. Unfortunately, we did not perform genotyping of PON Q192R patient in healthy Koreans, consequently we cannot prove this hypothesis.

### Limitation

There are several limitations in this study. First, we did not measure the PON activity in each patient. But several previous studies already reported that the PON activity is genetically controlled and 192Q-allele is associated with low activity [3], [15]. Second, there is a discrepancy in PON-1 Q192R allele frequency. The distribution of QQ, QR and RR genotypes were 35%, 47% and 18% in Bouman’s, 53%, 39%, 8% in Sibbing’s, 52%, 38%, 10% in Fontana’s study population. But in our study population, it was 13%, 48%, 39%, for QQ, QR and RR, respectively. The distribution of Q and R alleles differs in many populations. For example, the Q- and R-allele frequencies in Caucasian populations are 0.7 and 0.3, respectively, whereas this is inverted in African and Asian populations [30]. Therefore, our results cannot be directly extrapolated to Western populations, and vice versa. Third, all participants had significant CAD and received DES implantation. Therefore our findings are limited to the population analyzed in this study and cannot be generalized to others with mild CAD or those that do not receive PCI with DES. Most importantly, the low number of events in the study may raise the concern that the results may be by chance. Our results are hypothesis generating at best and further studies are needed to confirm our finding. Finally, since the LDL particle size analysis was performed at physicians’ discretion, patients at increased risk would more likely undergo LDL-particle analysis which is reflected by the differences in the baseline characteristics (**Table S2**).

### Conclusion

In patients receiving DES, PON1 Q-allele is an independent predictor of worse cardiovascular outcome independent of platelet function and is associated with significantly higher levels of small dense LDL-C. PON-1 genotype may serve as a novel genetic risk factor for adverse events after PCI.

## Supporting Information

Figure S1 **Kaplan Meier Survival analysis of composite of cardiac death, MI, ST according to genotypes.**
(TIF)

Figure S2 (TIF)

Table S1 **Baseline characteristics of the study population.**
(DOC)

Table S2 **Baseline characteristics of the study population according to LDL-particle size analysis.**
(DOC)

Table S3 **Clinical outcomes according to PON1-Q192R genotype.**
(DOC)

Table S4 **Lipid profile according to PON-1 Q192R genotype.**
(DOC)

Table S5 **Clopidogrel on-treatment platelet reactivity according to different loading regimen.**
(DOC)

Summary S1 (DOCX)
