# Supplementary figures and images for: Paraoxonase 1 Gene Polymorphism Does Not Affect Clopidogrel Response Variability but Is Associated with Clinical Outcome after PCI
Source: PLoS One. 2013 Feb 13;8(2):e52779. doi: 10.1371/journal.pone.0052779 (PMC3572125; doi:10.1371/journal.pone.0052779)

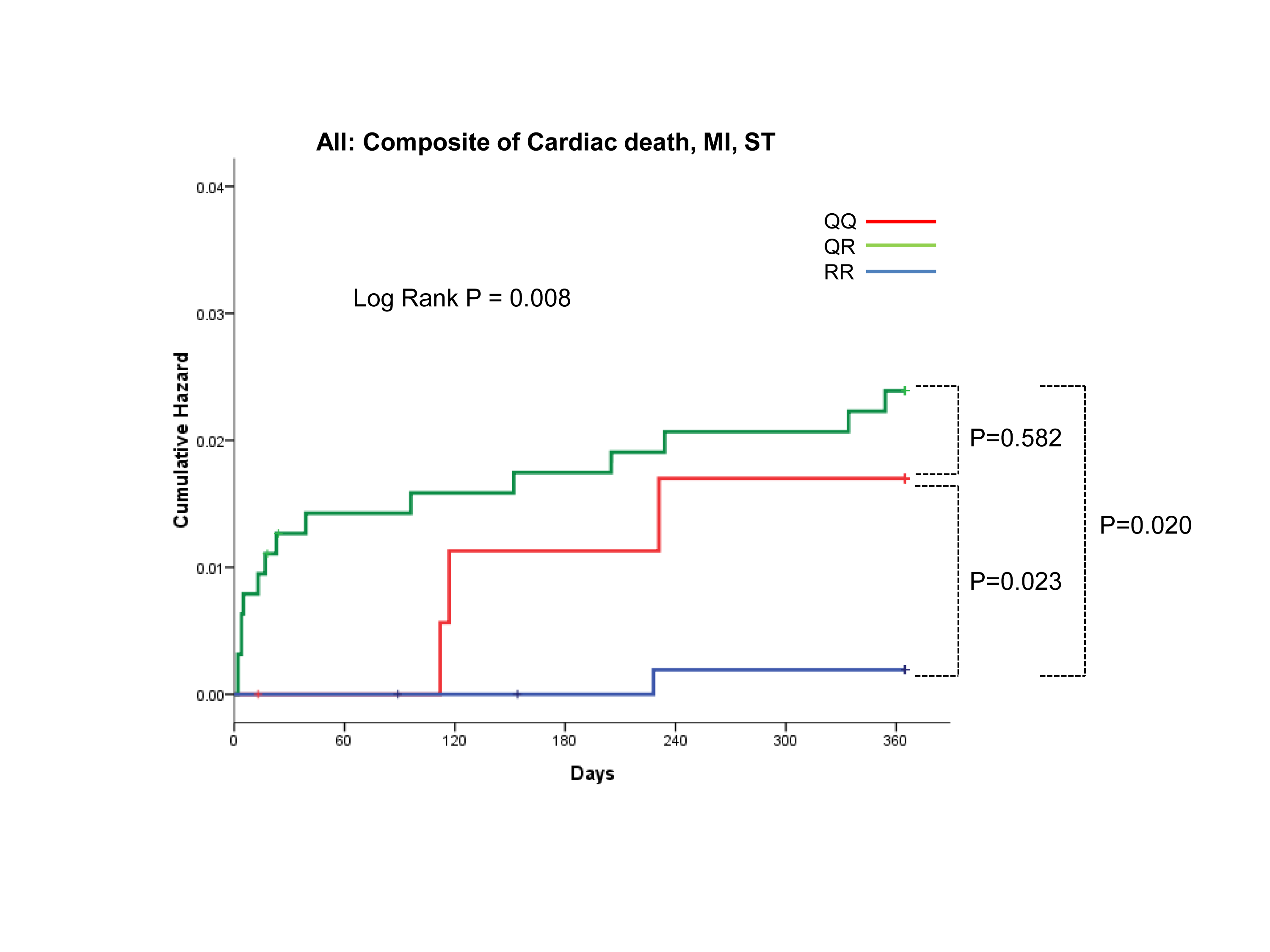

Supplement: Figure S1 — Kaplan Meier Survival analysis of composite of cardiac death, MI, ST according to genotypes. (TIF) [file pone.0052779.s001.tif]

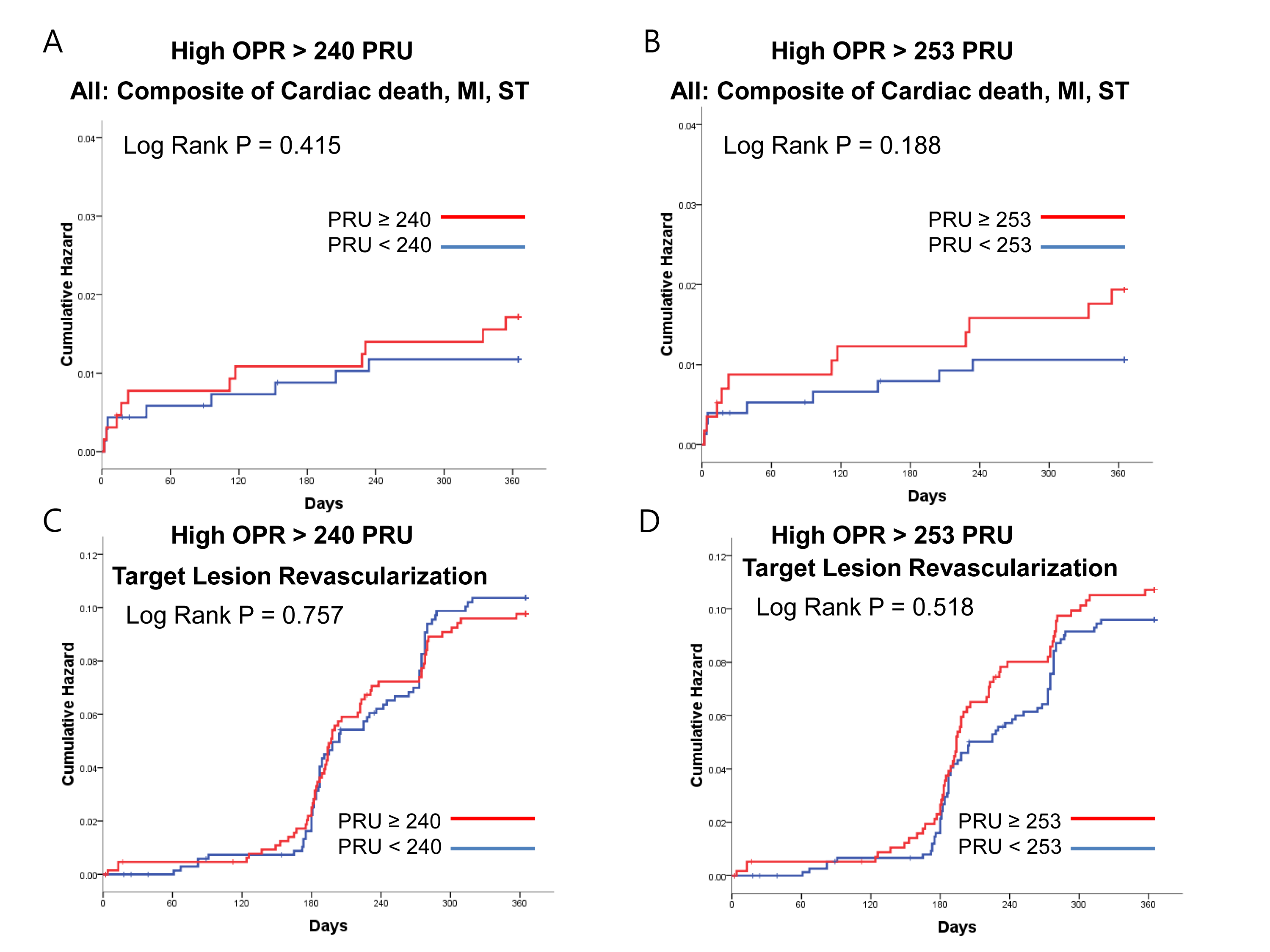

Supplement: Figure S2 — (TIF) [file pone.0052779.s002.tif]
