# Supplementary material for: Paraoxonase 1 Gene Polymorphism Does Not Affect Clopidogrel Response Variability but Is Associated with Clinical Outcome after PCI
Source: PLoS One. 2013 Feb 13;8(2):e52779. doi: 10.1371/journal.pone.0052779 (PMC3572125; doi:10.1371/journal.pone.0052779)
Supplement: Table S1 — Baseline characteristics of the study population. (DOC) [file pone.0052779.s003.doc]

**Table S1** Baseline characteristics of the study population

|  | All  N=1336 | QQ  (n=179) | QR  (n=636) | RR  (n=521) | p-value |
| --- | --- | --- | --- | --- | --- |
| **Demographic characteristics** | | | | | |
| Mean OPR (PRU) | 233±84 | 233±82 | 231±86 | 236±81 | 0.596 |
| Age (years) | 64±9 | 64±8 | 64±9 | 64±9 | 0.344 |
| Men (%) | 67.5 | 70.9 | 67.6 | 66.2 | 0.505 |
| Body mass index (kg/m^2^) | 25.2±4.8 | 24.7±2.9 | 25.0±3.0 | 25.5±6.8 | 0.081 |
| Current smoker (%) | 17.6 | 15.7 | 16.9 | 19.0 | 0.505 |
| Hypertension (%) | 67.6 | 68.7 | 66.0 | 69.1 | 0.511 |
| Diabetes mellitus (%) | 32.2 | 26.8 | 32.2 | 34.0 | 0.209 |
| Dyslipidemia (%) | 45.4 | 50.8 | 44.0 | 45.3 | 0.270 |
| Chronic kidney ds. (%) | 25.5 | 28.7 | 22.9 | 27.4 | 0.122 |
| - Renal replacement tx. | 1.0 | 0 | 1.1 | 1.2 | 0.361 |
| Presenting symptoms (%) |  |  |  |  | 0.418 |
| - Stable angina | 57.9 | 62.0 | 58.2 | 56.0 | 0.369 |
| - Unstable angina | 35.3 | 34.1 | 25.1 | 35.9 | 0.900 |
| - NSTEMI | 4.8 | 2.2 | 4.4 | 6.1 | 0.088 |
| - STEMI | 2.1 | 1.7 | 2.4 | 1.9 | 0.800 |
| Previous PCI (%) | 32.3 | 35.8 | 33.0 | 30.3 | 0.358 |
| Previous CABG (%) | 3.1 | 2.8 | 3.3 | 2.9 | 0.894 |
| Previous MI (%) | 4.6 | 5.6 | 4.2 | 4.8 | 0.735 |
| Congestive heart failure (%) | 0.7 | 0.6 | 0.9 | 0.6 | 0.733 |
| Cerebrovascular accident (%) | 5.8 | 4.5 | 5.3 | 6.7 | 0.443 |
| Peripheral artery disease (%) | 1.2 | 1.1 | 1.7 | 0.6 | 0.199 |
| **Laboratory finding** |  |  |  |  |  |
| GFR (ml/min/1.73m^2^) | 69.3±17.4 | 68.6±16.7 | 69.3±17.4 | 69.5±17.6 | 0.849 |
| Cholesterol (mg/dL) | 156±40 | 156±37 | 158±42 | 156±40 | 0.704 |
| - Triglyceride (mg/dL) | 143±94 | 143±83 | 137±82 | 149±110 | 0.109 |
| - HDL-C (mg/dL) | 43±12 | 42±10 | 42±11 | 43±14 | 0.714 |
| - LDL-C (mg/dL) | 88±36 | 85±31 | 90±37 | 87±36 | 0.124 |
| **Concomitant Medication** |  |  |  |  |  |
| ACEi/ARB (%) | 42 | 43.0 | 41.2 | 42.7 | 0.841 |
| Beta-blocker (%) | 51.1 | 53.1 | 51.3 | 50.2 | 0.793 |
| Calcium channel blocker (%) | 26.9 | 22.3 | 26.6 | 28.8 | 0.238 |
| -Dihydropyridine CCB (%) | 16.9 | 10.6 | 17.9 | 17.9 | 0.054 |
| -Non-DHP CCB (%) | 10.3 | 11.2 | 9.4 | 10.9 | 0.645 |
| Statin (%) | 62.5 | 62.6 | 63.1 | 61.8 | 0.909 |
| - Lipophilic statin (%) | 41.4 | 36.3 | 42.0 | 42.4 | 0.329 |
| Proton pump inhibitor (%) | 2.3 | 2.8 | 1.9 | 2.7 | 0.602 |
| - Omeprazol (%) | 0.8 | 0 | 0.3 | 1.7 | 0.146 |

ACEi, angiotensin converting enzyme inhibitor; ARB, angiotensin receptor blocker; CABG, coronary artery bypass graft; CCB, calcium channel blocker; HDL, high density lipoprotein; LDL, low density lipoprotein; OPR, on treatment platelet reactivity; PCI, percutaneous coronary intervention; PRU, P2Y12 reaction unit
