# Supplementary material for: Paraoxonase 1 Gene Polymorphism Does Not Affect Clopidogrel Response Variability but Is Associated with Clinical Outcome after PCI
Source: PLoS One. 2013 Feb 13;8(2):e52779. doi: 10.1371/journal.pone.0052779 (PMC3572125; doi:10.1371/journal.pone.0052779)
Supplement: Table S2 — Baseline characteristics of the study population according to LDL-particle size analysis. (DOC) [file pone.0052779.s004.doc]

**Table S2** Baseline characteristics of the study population according to LDL-particle size analysis

|  | All patients  N=1336 | LDL-particle size measurement  (n=769) | No LDL-particle size measurement (n=567) | p-value between LDL-particle size yes vs. no |
| --- | --- | --- | --- | --- |
| **Demographic characteristics** |  |  |  |  |
| Mean OPR (PRU) | 233±84 | 233±88 | 232±77 | 0.879 |
| Age (years) | 64±9 | 64.4±9.1 | 63.2±8.9 | 0.017 |
| Men (%) | 67.5 | 68.1% | 66.7% | 0.570 |
| Body mass index (kg/m^2^) | 25.2±4.8 | 25.4±5.8 | 24.9±3.0 | 0.113 |
| Current smoker (%) | 17.6 | 18.5% | 16.4% | 0.338 |
| Hypertension | 67.6 | 68.5 | 66.3 | 0.392 |
| Diabetes mellitus | 32.2 | 33.0 | 31.0 | 0.442 |
| Dyslipidemia | 45.4 | 44.7 | 46.4 | 0.549 |
| Chronic kidney disease | 25.5 | 23.5 | 28.1 | 0.057 |
| - Renal replacement tx. | 1.0 | 1.3 | 0.5 | 0.156 |
| Presenting symptoms |  |  |  | <0.001. |
| - Stable angina | 57.9 | 55.4 | 64.2 |  |
| - Unstable angina | 35.3 | 34.5 | 36.3 |  |
| - NSTEMI | 4.8 | 7.0 | 1.8 |  |
| - STEMI | 2.1 | 3.1 | 0.7 |  |
| Previous PCI | 32.3 | 29.0 | 36.9 | 0.002 |
| Previous CABG | 3.1 | 3.8 | 2.1 | 0.083 |
| Previous MI | 4.6 | 2.0 | 8.3 | <0.001 |
| Congestive heart failure | 0.7 | 0.9 | 0.5 | 0.531 |
| Cerebrovascular accident | 5.8 | 5.3 | 6.3 | 0.430 |
| Peripheral artery disease | 1.2 | 0.8 | 1.8 | 0.102 |
| **Laboratory finding** |  |  |  |  |
| GFR (ml/min/1.73m^2^) | 69.3±17.4 | 70.6±18.4 | 67.4±15.7 | 0.001 |
| Cholesterol (mg/dL) | 156±40 | 153.9±39.3 | 160.1±41.2 | 0.005 |
| - Triglyceride (mg/dL) | 143±94 | 139.9±94.9 | 146.8±93.7 | 0.189 |
| - HDL-C (mg/dL) | 43±12 | 42.4±12.7 | 42.7±11.0 | 0.695 |
| - LDL-C (mg/dL) | 88±36 | 85.8±34.1 | 91.4±37.3 | 0.005 |
| **Concomitant Medication** |  |  |  |  |
| ACEi/ARB | 42 | 49.1 | 32.5 | < 0.001 |
| Beta-blocker | 51.1 | 58.1 | 41.6 | < 0.001 |
| Calcium channel blocker | 26.9 | 28.9 | 24.2 | 0.055 |
| -Dihydropyridine CCB | 16.9 | 19.2 | 13.8 | 0.008 |
| -Non-DHP CCB | 10.3 | 10.2 | 10.4 | 0.882 |
| Statin | 62.5 | 80.6 | 37.9 | < 0.001 |
| - Lipophilic statin | 41.4 | 53.1 | 25.6 | < 0.001 |
| Proton pump inhibitor | 2.3 | 2.9 | 1.6 | 0.126 |
| - Omeprazol | 0.8 | 0.9 | 0 | 0.567 |

In our study, the LDL particle size analysis was performed at the physicians’ discretion and patients at increased risk would more likely undergo LDL-particle analysis which is reflected by the differences in the baseline characteristics. Therefore, all analyses were performed with adjustment for statin-use along with age, gender, TG, HDL-C.
