# Supplementary material for: Paraoxonase 1 Gene Polymorphism Does Not Affect Clopidogrel Response Variability but Is Associated with Clinical Outcome after PCI
Source: PLoS One. 2013 Feb 13;8(2):e52779. doi: 10.1371/journal.pone.0052779 (PMC3572125; doi:10.1371/journal.pone.0052779)
Supplement: Table S3 — Clinical outcomes according to PON1-Q192R genotype. (DOC) [file pone.0052779.s005.doc]

**Table S3 Clinical outcomes according to PON1-Q192R genotype**

|  | QQ  n=179,  13.4% | QR  N=636,  47.6% | RR  n=521,  39.0% | Log-rank  p-value |
| --- | --- | --- | --- | --- |
| **Primary composite end point of cardiac death, MI, and ST** | 3 (1.7%) | 15 (2.4%) | 1 (0.2%) | 0.008* |
| **Secondary end points** |  |  |  |  |
| Cardiac Death | 1 (0.6%) | 7 (1.1%) | 1 (0.2%) | 0.167 |
| Myocardial infarction | 3 (1.7%) | 8 (1.3%) | 0 (0%) | 0.025 |
| CVA | 0 (0%) | 1 (0.2%) | 0 (0%) | 0.577 |
| Stent thrombosis | 1 (0.6%) | 7 (1.1%) | 0 (0) | 0.054 |
| Target lesion revascularization | 18 (10.1%) | 62 (9.7%) | 47 (9.0%) | 0.883 |
| Composite of Death, MI, CVA | 4 (2.2%) | 15 (2.4%) | 1 (0.2%) | 0.007 |
| MACE | 2 (14.5%) | 87 (13.7%) | 54 (10.4%) | 0.161 |

* Log-rank P-value for all groups, QQ vs. QR, Log Rank P = 0.582, QQ vs. RR, Log Rank P = 0.023, QR vs. RR, log rank P = 0.020

MI, myocardial infarction; ST, stent thrombosis; CVA; cerebrovascular accident; MACCE, major adverse cardiovascular event

P-value by Log-rank test
