# Supplementary material for: Paraoxonase 1 Gene Polymorphism Does Not Affect Clopidogrel Response Variability but Is Associated with Clinical Outcome after PCI
Source: PLoS One. 2013 Feb 13;8(2):e52779. doi: 10.1371/journal.pone.0052779 (PMC3572125; doi:10.1371/journal.pone.0052779)
Supplement: Table S4 — Lipid profile according to PON-1 Q192R genotype. (DOC) [file pone.0052779.s006.doc]

**Table S4** Lipid profile according to PON-1 Q192R genotype

|  | QQ/QR | RR | p-value |
| --- | --- | --- | --- |
| Cholesterol (md/dl) | 157±41 | 156±40 | 0.488 |
| Triglyceride (md/dl) | 139±82 | 149±110 | 0.047 |
| HDL (md/dl) | 42±11 | 43±14 | 0.412 |
| LDL (md/dl) | 89±36 | 87±36 | 0.203 |
| **LDL-particle size** |  |  |  |
| - VLDL (md/dl) | 25.04±0.34 | 24.23±0.42 | 0.131 |
| - ILD1 (md/dl) | 13.65±0.22 | 13.00±0.28 | 0.052 |
| - IDL2 (md/dl) | 11.27±0.19 | 11.01±0.24 | 0.386 |
| - IDL3 (md/dl) | 12.33±0.22 | 12.03±0.27 | 0.386 |
| - LDL-I (md/dl) | 27.23±0.47 | 27.05±0.59 | 0.813 |
| - LDL-IIA (md/dl) | 19.85±0.46 | 19.61±0.58 | 0.748 |
| - LDL-IIB (md/dl) | 5.50±0.27 | 4.74±0.33 | 0.075 |
| - LDL-IIIA (md/dl) | 1.20±0.12 | 0.76±0.15 | 0.027 |
| - LDL-IIIB (md/dl) | 0.22±0.05 | 0.19±0.07 | 0.702 |
| - LDL-IVA (md/dl) | 0.06±0.03 | 0.08±0.03 | 0.453 |
| - LDL-IVB (md/dl) | 0.03±0.10 | 0.31±0.13. | 0.091 |

As for LDL-particle size, analysis of covariance (ANCOVA) was applied to quantify the effect of PON-1 QQ/QR genotype on LDL-particle size after adjustment for age, sex, triglyceride level, HDL-C level, and statin use. Data were presented as mean ± SEM.
