# Supplementary material for: Paraoxonase 1 Gene Polymorphism Does Not Affect Clopidogrel Response Variability but Is Associated with Clinical Outcome after PCI
Source: PLoS One. 2013 Feb 13;8(2):e52779. doi: 10.1371/journal.pone.0052779 (PMC3572125; doi:10.1371/journal.pone.0052779)
Supplement: Table S5 — Clopidogrel on-treatment platelet reactivity according to different loading regimen. (DOC) [file pone.0052779.s007.doc]

**Table S5** Clopidogrel on-treatment platelet reactivity according to different loading regimen.

|  | QQ | QR | RR | P-value |
| --- | --- | --- | --- | --- |
| All-patients (n=1446) | 233±82 | 231±86 | 236±81 | 0.596 |
| Chronic-users (n=659, 49.3%) | 242±85 | 231±84 | 236±82 | 0.516 |
| Loading (n=677, 50.7%) | 224±79 | 230±88 | 236±82 | 0.535 |
| - 300-mg loading dose (52.6%) | 237±63 | 238±83 | 246±77 | 0.653 |
| - 600-mg loading dose (47.4%) | 219±99 | 218±89 | 234±83 | 0.856 |
