# Supplementary material for: Paraoxonase 1 Gene Polymorphism Does Not Affect Clopidogrel Response Variability but Is Associated with Clinical Outcome after PCI
Source: PLoS One. 2013 Feb 13;8(2):e52779. doi: 10.1371/journal.pone.0052779 (PMC3572125; doi:10.1371/journal.pone.0052779)
Supplement: Summary S1 — (DOCX) [file pone.0052779.s008.docx]

**Supporting Information Summary**

**I. Baseline characteristics of the study population**

**Table S1.** Baseline characteristics of the study population.

**Table S2.** Baseline characteristics of the study population according to LDL-particle size analysis.

**II. Clinical outcome according to PON Q912R genotypes by using additive genetic model**

Since the clinical significance of PON-Q195R genotype is not well understood, and there exists no consensus way to group these genotypes based on mode of inheritance, moreover, different grouping might have resulted in different outcomes.

Therefore, we undertook further analysis to see the clinical outcomes according to the three genotypes, i.e. QQ, QR and RR-genotypes. The primary composite endpoint of cardiac death, MI, and stent thrombosis occurred in 3 patients (1.7%) in QQ, 15 patients (2.4%) in QR, and 1 (0.2%) in RR-genotypes (Log rank p = 0.008) (**Table S3**).

When comparing the outcomes between the different genotypes, there was no difference in event rate between QQ and QR genotypes (Log-rank P = 0.582), whereas significant differences were observed between QQ vs. RR (Log-rank P = 0.023), and QR and RR (Log-rank P = 0.020), suggesting a similar “clinical behavior” of Q-allele carriers (**Figure S1**).

**Table S3** Clinical outcomes according to PON1-Q192R genotype

**Figure** S**1** Kaplan Meier Survival analysis of composite of cardiac death, MI, ST according to genotypes.

**III. Lipid profile**

**Table S4** Lipid profile according to PON-1 Q192R genotype

**IV. Clopidogrel response variability according to dosing regimen**

The platelet response to clopidogrel is heterogeneous and the dosing regimen and dosing timing can exert significant effect on clopidogrel response-variability. Therefore we undertook further analysis according to the clopidogrel dosing regimen and platelet reactivity according to PON-1 Q192R genotypes.

The OPR of QQ, QR, and RR genotypes in whole population were 233±82 PRU, 231±86 PRU, and 236±81 PRU, for QQ, QR, and RR genotypes, respectively (ANOVA P = 0.596). Among them, 659 patients (49.3%) were on chronic clopidogrel administration, whereas 677 patients (50.7%) received a clopidogrel loading dose. There was no difference in clopidogrel OPR between the three genotypes in chronic users (mean OPR: 242±85 PRU, 231±84 PRU, and 236±82 PRU, for QQ, QR, and RR genotypes, respectively; ANOVA P = 0.516), as well as in patients who received a loading dose (mean OPR: 224±79 PRU, 230±88 PRU, and 236±82 PRU, for QQ, QR, and RR genotypes, respectively; ANOVA P = 0.535). The results were similar when comparing the clopidogrel OPR according to 300-mg and 600-mg loading dose (**Table S5**).

**Table S5** Clopidogrel on-treatment platelet reactivity according to different loading regimen.

**V. Clopiodgrel response variability according to CYP2C19 genotype**

Clopidogrel is a prodrug that needs to be converted into active metabolites by hepatic cytochrome P450 enzymes (CYP) in two oxidation steps. Among the CYP-enzymes, the CYP2C19 *2 and *3 loss of function (LOF) polymorphisms have been consistently associated with high OPR.

We genotyped the CYP2C19 *2, and *3, and the results of 1,328 patients were available for the analysis. The frequencies of genotypes for *1/*1, *1/*2, *1/*3, *2/*2, *2/*3, *3/*3 were 40.4%, 33.4%, 15.7%, 8.3%, 0.7%, and 1.6%, respectively. Patients with *1/*2 and *1/*3 classified to have 1 LOF-allele, and patients with *2/*2, *2/*3, *3/*3 were classified to have 2 LOF-alleles. The frequencies for patients with 0 LOF-allele, 1 LOF-allele and 2 LOF-alleles were 40.4%, 49.1% and 10.5%.

The OPR increased significantly from 0 to 2 LOF alleles (215±81 PRU for 0-LOF, 241±84 PUR for 1 LOF, 265±74 PRU for 2 LOF, ANOVA P <0.001).

**VI. Clopidogrel response variability and clinical outcomes**

Clopidogrel is an important component of antiplatelet therapy, and has proven benefit in primary and secondary prevention in large clinical trials. Inter-individual and inter-racial differences in clopidogrel response have been, and high residual platelet aggregation has been associated with increased thrombotic complications.

We used two cut-off values to define high-on treatment platelet reactivity: a cutoff value of 240 PRU derived from Caucasians (Patti et al, Circulation 2011), and a cutoff value of 253 PRU derived from Asians (Suh et al, JACC 2011).

As for the cutoff value of 240 PRU, 648 patients (48.5%) had high-OPR. The rate of composite primary endpoint of cardiac death, MI, and stent thrombosis was numerically higher in high-OPR group (11/648=1.7%) than normal-OPR group (8/687=1.2%), however without statistical significance (Log-rank P = 0.415) (**Figure S2A**). With regard to TLR, there was no difference between both groups (normal-OPR: 9.8% vs. high-OPR: 9.3%, Log-rank P = 0.757) (**Figure S2C**).

As for cutoff value of 253 PRU, 574 patients (43%) had high-OPR, and they had numerically higher rate of primary endpoint (high OPR: 11/574 = 1.9% vs. normal OPR: 8/761 = 1.1%), but without statistical significance (Log-rank P = 0.188) (**Figure S2B**). When stratifying the patients according to the clinical presentation, in patients with stable angina, the primary composite endpoint was higher in high-OPR group (high-OPR: 7/332=2.1% vs. normal-OPR: 2/442=0.5%, Log-rank P = 0.034), whereas no difference was observed among ACS patients. With regard to TLR, there was no difference between both groups, either (normal-OPR: 9.1% vs. high-OPR: 10.1%, Log-rank P = 0.518) (**Figure S2D**). Although there was no statistically significant difference in event rate of primary composite endpoint between high-OPR and normal-OPR group, the number of events was higher in high-OPR group.
